# Hyponatremia in Patients with Hematologic Diseases

**DOI:** 10.3390/jcm9113721

**Published:** 2020-11-19

**Authors:** Epameinondas Koumpis, Matilda Florentin, Eleftheria Hatzimichael, George Liamis

**Affiliations:** 1 Department of Hematology, Faculty of Medicine, University of Ioannina, Stavros Niarchos Avenue, GR-45110 Ioannina, Greece; ekoumpis@outlook.com (E.K.); ehatzim@uoi.gr (E.H.); 2 Department of Internal Medicine, Faculty of Medicine, University of Ioannina, Ioannina, Stavros Niarchos Avenue, GR-45110 Ioannina, Greece; matildaflorentin@yahoo.com

**Keywords:** hyponatremia, hematology, sodium, cancer, drugs, SIADH, RSWS

## Abstract

Hyponatremia is the most common electrolyte disorder in clinical practice and is associated with increased morbidity and mortality. It is frequently encountered in hematologic patients with either benign or malignant diseases. Several underlying mechanisms, such as hypovolemia, infections, toxins, renal, endocrine, cardiac, and liver disorders, as well as the use of certain drugs appear to be involved in the development or the persistence of hyponatremia. This review describes the pathophysiology of hyponatremia and discusses thoroughly the contributing factors and mechanisms that may be encountered specifically in patients with hematologic disorders. The involvement of the syndrome of inappropriate antidiuretic hormone (SIADH) secretion and renal salt wasting syndrome (RSWS) in the development of hyponatremia in such patients, as well as their differential diagnosis and management, are also presented. Furthermore, the distinction between true hyponatremia and pseudohyponatremia is explained. Finally, a practical algorithm for the evaluation of hyponatremia in hematologic patients, as well as the principles of hyponatremia management, are included in this review.

## 1. Introduction

Hyponatremia, usually defined as serum sodium concentration < 135 mEq/L, is the most common electrolyte abnormality encountered both in hospitalized patients and in the general population and is associated with increased morbidity and mortality [1,2]. The rapidity and the degree of reduction in serum sodium concentration are the main determinants of the symptoms associated with this electrolyte disorder. In the setting of acute (<48 h) or severe (serum sodium levels < 120 mEq/L) hyponatremia, patients’ symptoms may range from non-specific nausea, vomiting, and headache to life-threatening stupor, coma, seizures, respiratory depression, and death. Chronic moderate (serum sodium levels 120–129 mEq/L) and mild (serum sodium levels 130–134 mEq/L) hyponatremia are usually asymptomatic and have no remarkable findings on a conventional clinical examination. However, individuals with such sodium levels may develop subtle manifestations, e.g., fatigue, cognitive impairment, disorientation and gait disorders, as well as falls, osteoporosis, and fractures [3,4]. The incidence of hyponatremia varies extensively in different studies mainly depending on the definition of hyponatremia and the patient population. Thus, an incidence of 7.2% has been reported in the general population, while it may reach up to 42.6% in hospitalized patients [5].

Hyponatremia may be encountered in several hematological diseases, both benign and malignant [6,7]. In a study including hospitalized children treated for acute lymphoblastic leukemia (ALL), the incidence of hyponatremia (serum sodium levels < 130 mmol/L) on at least 2 out of 3 consecutive days was 11.9% [8]. Moreover, hyponatremia was significantly related both to neurologic complications and the existence of obvious central nervous system leukemia at diagnosis [8]. In another single center analysis of 140 pediatric patients, hyponatremia was observed in 40% of patients following hematopoietic stem cell transplantation (HSCT) [9]. A large retrospective cohort analysis of patients diagnosed with particular cancer types showed that, in patients with lymphoma, the incidence rate of euvolemic and hypervolemic hyponatremia was 395 per 1000 person-years [10]. Of note, one recent experimental study illustrated that low extracellular sodium concentration may promote carcinogenesis in vitro by upregulating molecular pathways involved in oxidative stress, proliferation, and invasion [11].

The importance of recognizing, evaluating, and treating hyponatremia in hematologic patients lies in the fact that it is an independent predictor of unfavorable outcomes both in patients with neoplastic and benign disorders, including lymphomas, sickle cell anemia, hemolytic uremic syndrome, and allogeneic hematopoietic SCT (AlloSCT) [7,10,12,13]. In addition, symptoms of hyponatremia such as fatigue, disorientation, or even falls can be mistakenly attributed to other causes, such as neutropenic sepsis or central nervous system (CNS) involvement in the context of the underlying hematological disease. This review discusses the causes as well as the clinical and pathophysiological aspects of hyponatremia in the spectrum of hematological disorders. The proper treatment of hyponatremia is also presented.

## 2. Pathophysiology

The two main pathophysiological mechanisms of hyponatremia include either loss of effective solutes (sodium plus potassium) in excess of water or (more often) water retention. Given that the ability for water excretion is sufficient in normal states, retention of water resulting in reduced serum sodium concentration occurs only in the presence of impaired renal excretion of water. Primary polydipsia represents an exception to this rule, in which the disproportionate water intake can overwhelm the normal excretory capacity (acute water intoxication) [14,15].

High serum levels of arginine vasopressin (also known as antidiuretic hormone (ADH)) should be considered as a prerequisite for the development and maintenance of hyponatremia in view of the fundamental role of the suppression of ADH secretion for the renal excretion of any water load. Thus, irrespective of the presence of hypotonicity, almost all causes of hyponatremia (except for low dietary solute intake, renal failure, primary polydipsia, or beer potomania syndrome) are accompanied by increased ADH, mainly due to the syndrome of inappropriate ADH secretion (SIADH) or to effective circulating volume depletion. In fact, a 15% decrease in effective arterial blood volume due to true hypovolemia (e.g., vomiting, diarrhea, osmotic diuresis) or edematous states (e.g., congestive heart failure, nephrotic syndrome, hepatic cirrhosis with ascites) causes a reduction in stretch at the carotid and renal baroreceptors, subsequently increasing ADH excretion and overriding the inhibitory effect of hypotonicity. This emphasizes the greater importance of mechanisms aiming at the maintenance of adequate circulating volume at the expense of osmotic dysregulation and hyponatremia [16].

The volume of ingested water and daily solute intake also play an important role in the development of hyponatremia. Although the kidneys have a great ability to excrete large amounts of water, this is not unlimited even in the case of intact diluting capacity. The daily urine volume (UV) is calculated by the following equation: UV = USL/Uosm, where USL is the urine solute load (in mOsm/day) and Uosm is the urine osmolarity. A normal Western diet provides 600–900 mOsm of solute daily, derived mainly from urea (the metabolic product of proteins) and electrolytes (sodium, potassium, and accompanying anions), which generates an equivalent USL. Thus, at a lowest achievable Uosm (50 mOsm), 18 L of urine will be excreted if solute intake is 900 mOsm/d. On the contrary, only 2 L of urine can be excreted if the intake of solutes is reduced to as low as 100 mOsm/d, an amount not infrequently observed in malnourished patients (tea and toast diet). As a result, if these patients consume more than 2 L of fluids, hyponatremia will ensue [17,18].

## 3. Causes of Hyponatremia in Hematologic Patients

### 3.1. Pseudohyponatremia

The first step when evaluating hyponatremia is to exclude the possibility of pseudohyponatremia; this may result from marked hyperlipidemia or hyperproteinemia (the causes of hyperlipidemia or hyperproteinemia in patients with hematologic diseases are shown in Table 1 and discussed below)—conditions that reduce the water content of a given volume of serum. Hence, sodium concentration measured per liter of serum is artifactually decreased (pseudohyponatremia), while sodium concentration in the water phase and serum osmolality are not affected. Electrolytes can be measured via direct or indirect ion-selective electrodes (ISE). Only indirect ISE is related to spurious hyponatremia, as it requires a dilution step [19]. Therefore, the obstacle of pseudohyponatremia may be avoided with direct ISE.

Hypocholesterolemia is the most common lipid abnormality in patients with cancer, including hematologic malignancies [20]. However, hyperlipidemia may also occur. A case of type III hyperlipoproteinemia with xanthomas has been reported in a patient with multiple myeloma (MM) [21]. Furthermore, severe secondary hypercholesterolemia due to cholestasis in a patient who had obstructive jaundice as the initial presentation of non-Hodgkin’s lymphoma (NHL) has been reported [22]. L-asparaginase, an essential agent in treating ALL, induces hypertriglyceridemia, which may be further intensified by the co-administration of steroids [23,24]. Furthermore, all trans retinoic acid (ATRA) used to treat acute promyelocytic leukemia appears to cause dose-dependent hypertriglyceridemia [25]. AlloSCT may also lead to hypercholesterolemia either due to decreased activity of hepatic triglyceride lipase or due to cholestasis induced by chronic graft versus host disease (GvHD) [26,27]. Furthermore, hypertriglyceridemia occurs with hemophagocytic lymphohistiocytosis (HLH) and, in fact, represents one of its diagnostic criteria [28,29].

Importantly, several conditions encountered in hematologic patients are accompanied by nephrotic syndrome and, thus, are probable causes of spurious hyponatremia in the context of hyperlipidemia (Table 1). The main underlying mechanisms of nephrotic syndrome-related hyperlipidemia are the increased hepatic biosynthesis as well as the decreased clearance of cholesterol and major lipoproteins, triggered by the decrease in oncotic pressure due to loss of proteins in urine [30,31,32,33].

Hepatitis B and C viruses (HBV and HCV) are common infections in patients with hemoglobinopathies, such as thalassemias, due to repetitive transfusions [34,35]. These infections have been associated with nephrotic-syndrome- or cholestasis-related hyperlipidemia as well as hypergammaglobulinemia due to chronic liver disease; thus, pseudohyponatremia may occur [36,37]. Monoclonal gammopathy and intravenous immunoglobulin (IVIG) administration have also been associated with spurious hyponatremia [38,39]. In the former, pseudohyponatremia may occur in the setting of severe hyperproteinemia (usually greater than 10 g/dL), given that a 1 g/dL increase in serum protein concentration decreases serum sodium concentration by approximately 0.7 mEq/L [40]. In the latter, both pseudohyponatremia and true hyponatremia may be observed (see below).

It should be emphasized that normal serum sodium levels in the context of hyperproteinemia or hyperlipidemia should raise the suspicion of hypernatremia (pseudonormonatremia). Moreover, in hypoalbuminemic states (e.g., nephrotic syndrome), indirect ISE may overestimate serum sodium concentration up to 10 mEq/L compared with direct ISE [41]. Therefore, in the presence of hyperlipidemias, hyper- and hypoalbuminemia serum sodium levels should be measured with the direct ISE method, while in such cases, it is prudent to measure serum osmolality by an osmometer. The presence of pseudohyponatremia is ascertained when the measured serum osmolality is within normal limits (280–295 mOsm/kg) [19].

### 3.2. SIADH

SIADH is one of the most common causes of hyponatremia and may be attributed to numerous underlying conditions. Although inappropriate release of ADH is a requirement for this condition, increased intake of liquids also plays an essential role in developing low serum sodium concentrations [42]. When SIADH occurs in the context of malignant hematologic diseases (e.g., lymphomas, leukemia, MM, Waldenström’s macroglobulinemia (WM)), it is mainly ascribed to ectopic ADH secretion or increased interleukin-6 (IL-6) production from malignant cells, as well as to CNS infiltration [43,44,45,46,47,48].

IL-6 induces SIADH by increasing (non-osmotically) the hypothalamic production of ADH [49]. IL-6-mediated SIADH has also been observed in hemophagocytic syndrome [50]. Increased serum levels of lead have also been suggested as an etiologic factor of SIADH in a 5-year-old patient with sickle cell disease, where both hyponatremia and increased levels of ADH were corrected by dimercaprol and calcium ethylenediaminetetraacetic acid (EDTA) chelation treatment [51]. However, it appears more plausible that high levels of IL-6 and stroke due to acute vaso-occlusion play the most important role in SIADH associated with sickle cell disease [52].

SIADH has also been reported in acute intermittent porphyria and post AlloSCT [13,53]. Of note, early presentation of hyponatremia due to SIADH post AlloSCT is insidious and may progress rapidly with fatal outcome [13]. SIADH following AlloSCT or even autoSCT is a relatively infrequent and poorly recognized disorder. Non-specific symptoms of hyponatremia such as nausea, vomiting, and fatigue may be easily attributed to the conditioning regimen prior to transplant. Several risk factors have been implicated in the presentation of SCT-related SIADH, including among others cord blood as the source of the graft, human leukocyte antigen (HLA)-mismatched unrelated donor or recipient, age below 4 years [9], cyclophosphamide [54] or busulphan as components of the conditioning regimen [55], and GvHD prophylaxis with methylprednisolone [9] or tacrolimus [56]. Furthermore, cord blood as the source of the graft, compared to peripheral blood or bone marrow, has been correlated with more severe symptoms, e.g., seizures, somnolence, and earlier onset of hyponatremia [57].

IL-6 and TNF-a have been implicated in the pathophysiology of SCT-related SIADH. These cytokines have been reported to be elevated post SCT from an HLA-mismatched donor or unrelated donor [58]. However, no correlation has been found between acute GvHD and SCT-related SIADH [9,57] as one would expect. Another interesting mechanism of SCT-related SIADH is the reactivation of varicella zoster virus (VZV), one of the most common post-transplant complications with atypical manifestations [59,60,61]. Rau et al. suggested that the triad of severe abdominal pain, inappropriate ADH secretion, and disseminated VZV infection preceding skin lesions should prompt clinicians for VZV DNA blood detection for early recognition and treatment [62].

Other infections affecting the respiratory or CNS as well as several drugs (discussed below) are also frequent underlying causes of SIADH in hematologic diseases. Moreover, pain, nausea, and stress, which are frequently encountered in hematologic patients, are non-osmotic stimuli for ADH release [42].

### 3.3. Hypovolemia

Extracellular volume depletion is among the most common causes of hyponatremia in clinical practice. Hematologic patients may develop hypovolemic hyponatremia due to renal or extrarenal fluid losses induced by infections (see below), drugs (e.g., vomiting or diarrhea associated with chemotherapy), or the underlying hematologic disease per se. A rather uncommon cause of volume depletion is cerebral salt wasting syndrome (CSWS), first described by Peters JP et al. in 1950 [63]. The release of brain natriuretic peptide leading to natriuresis and hypovolemia has been suggested as the major pathophysiologic mechanism of CSWS [64,65]. Myeloproliferative diseases have been associated with CSWS; it is likely that these disorders cause ischemic lesions in the brain via hyperviscosity and microcirculating abnormalities [66]. CSWS-induced hyponatremia has been observed after AlloSCT and was accompanied by CNS complications (e.g., cerebral hemorrhage, encephalitis) [67], as well as in sickle cell disease [68]. Another term for CSWS is renal salt wasting syndrome (RSWS), given that the presence of cerebral disease is not necessary [69]. Hyponatremia due to renal salt loss ascribed to oncolysis-induced cytokine release has been reported in a patient suffering from natural killer-cell neoplasm accompanied by hemophagocytic syndrome [70]. Salt-losing nephropathy has also been attributed to leukemia-induced tubular defect [47]. In addition, polyuria may occur in central diabetes insipidus (due to leukemia or lymphoma) [71,72] or in nephrogenic diabetes insipidus (due to sickle cell disease or trait and renal amyloidosis) [73,74].

### 3.4. Hyponatremia Related to Infections in Hematology

Infections, which frequently complicate the clinical course of hematological patients, may cause hyponatremia with several different mechanisms [75]. During the course of an infection, diarrhea, vomiting, or excessive sweating may occur, leading to hypovolemic hyponatremia. Of note, certain infections may be triggered by several treatments used in hematologic patients (e.g., chemotherapy) in combination with the reduced underlying humoral or cell-mediated immunity. For example, in a patient treated with chemotherapy for follicular lymphoma (FL), diarrhea due to severe cytomegalovirus colitis contributed to hyponatremia [76]. Symptomatic hypovolemic hyponatremia ascribed to dengue hemorrhagic fever has also been reported in patients with thalassemias who are susceptible to this infection (endemic in Southeast Asia) [77].

SIADH due to increased hypothalamic production of ADH frequently complicates the course of viral, bacterial, fungal, and tuberculous infections affecting mainly the lungs and the CNS [75]. As aforementioned, acquired hemophagocytic syndrome, which can be triggered by infections (mostly viral), may cause IL-6-mediated SIADH [50]. Of note, coronavirus disease 2019 (COVID-19), which seems to affect the hematopoietic system (e.g., lymphopenia, coagulopathy) has also been associated with IL-6-related hyponatremia [78,79]. Other causes of hyponatremia in the context of an infection are primary (e.g., systemic fungal infections, acquired immunodeficiency syndrome) or secondary adrenal insufficiency (e.g., tuberculosis), severe renal injury (e.g., leptospirosis), nephrotic syndrome (e.g., HBV and HCV), CSWS (e.g., cerebral toxoplasmosis or human herpesvirus 6 encephalitis), and congestive heart failure (infection-induced myocarditis) [75]. Furthermore, by increasing the secretion of catecholamines, glucagon, and cortisol, infections may promote hyperglycemia and, subsequently, hyponatremia (see next section).

Importantly, some antibiotics may induce hyponatremia. Trimethoprim, which structurally resembles the potassium-sparing diuretic amiloride, may cause hypovolemic hyponatremia, especially if administered in high doses [75]. Other antibiotics or antifungals (e.g., ciprofloxacin, pentamidine, voriconazole) are rarely implicated in the development of hyponatremia [75].

Several infections (e.g., infective endocarditis, leishmaniasis, human immunodeficiency virus (HIV) infection, HCV) through polyclonal activation of B-lymphocytes may induce hypergammaglobulinemia and, accordingly, pseudohyponatremia [75]. Noteworthy, most of the aforementioned pathophysiological pathways causing hyponatremia may be observed in HIV infection [75]. Low serum sodium concentration in the context of an infection may be a negative prognostic indicator of mortality; in fact, in an observational, retrospective, cross-sectional study in patients < 18 years old with Shiga toxin-producing Escherichia coli hemolytic uremic syndrome, hyponatremia was a mortality predictor [7].

### 3.5. Hyponatremia Due to Disorders of Endocrine System and Metabolism in Hematology

Diabetes mellitus (DM) is frequently observed in the adult general population, and its incidence is constantly rising having reached epidemic proportions. Noteworthy, a meta-analysis of observational studies demonstrated that DM may increase the risk of developing non-Hodgkin lymphoma, leukemia, and myeloma [80]. Certain hematologic disorders (e.g., hemochromatosis, major thalassemia) are pathophysiologically linked to DM. Specifically, it has been postulated that DM is the result of iron overload, the latter being deposited to the pancreas and causing oxidative stress to β-cells, subsequently leading to pancreatic dysfunction [81,82]. DM as a complication of major thalassemia is usually manifested after the first decade of life [82,83]. Hyperglycemia in hematological patients may also be the result of infections or certain medications [84,85,86]. For instance, corticosteroids are frequently used for the treatment of both malignant and benign (e.g., immune thrombocytopenia and autoimmune hemolytic anemia) disorders. Furthermore, immunosuppressive drugs, such as tacrolimus, have been associated with post-transplant DM [86]. Long-term survivors of HSCT are at increased risk of developing metabolic syndrome and DM; these probably ensue in the context of the long-term effects of intensive chemotherapy, as well as the immunological and inflammatory consequences of GvHD and its treatment [87]. Immune checkpoint inhibitors, such as pembrolizumab and nivolumab used in Hodgkin’s lymphoma (HL), have been associated with autoimmune diabetes and even diabetic ketoacidosis [88]. Finally, interferon therapy has been associated with hyperglycemia and DM [89].

Glucose is an osmotically active substance; thus, in the context of hyperglycemia, osmotic shifts of water from the intracellular to the extracellular space lead to dilutional hyponatremia. In hyperglycemic states, serum sodium concentration should be corrected; the most commonly used formula is corrected sodium = measured sodium + (1.6 (glucose − 100)/100). When glucose concentration is above 400 mg/dL, a correction factor of 2.4 should be used. Poorly controlled DM may cause hypovolemic hyponatremia via osmotic diuresis, whereas in patients with diabetic ketoacidosis, the excretion of beta-hydroxybutyrate and acetoacetate aggravates urine sodium losses [90]. Of note, DM per se (in the absence of hyperglycemia) may lead to hyponatremia, possibly through induction of aquaporin-2 (AQP2) by insulin [91]. AQP2 is found in the apical cell membranes of the principal cells in the collecting duct of the kidney, as well as in intracellular vesicles located throughout these cells. It is regulated by vasopressin and is involved in water reabsorption. Thus, in the case of increased expression of AQP2, excessive water reabsorption and subsequently hyponatremia occur [92].

Other endocrine disorders, including primary adrenal insufficiency (Addison’s disease), secondary adrenal insufficiency, and hypothyroidism, are less frequent causes of hyponatremia [16]. The association between infections and adrenal insufficiency in hematologic individuals has already been discussed.

Hyponatremia has been observed in patients with intravascular large B-cell lymphoma both via SIADH and hypopituitarism [93,94]. On the other hand, diffuse large B cell lymphoma (DLBCL) may lead to hyponatremia through bilateral adrenal or hypothalamus invasion [95,96]. In fact, a recent meta-analysis showed that malignancies, including lymphomas, may lead to adrenal insufficiency due to bilateral adrenal infiltration [97]. A case of bilateral adrenal hemorrhage in a patient with acute myeloid leukemia (AML) has also been reported [98]. Furthermore, adrenal insufficiency may be the result of isolated adrenocorticotropic hormone (ACTH) deficiency in ALL patients [99]. Patients with hemoglobinopathies, including thalassemias and sickle cell disease, may present with several endocrine disorders, including hypogonadotropic hypogonadism, DM, hypothyroidism, hypoparathyroidism, and adrenal insufficiency, mainly due to iron overload [100,101]. Adrenal insufficiency due to plasma cell infiltration with light-chain (AL) amyloid deposition in the pituitary and adrenal gland has been reported in a patient with WM [46].

Adrenal insufficiency may occur due to inappropriate interruption of glucocorticoid therapy, as the hypothalamus adrenal axis is suppressed. This, however, may be easily prevented by meticulous explanation regarding corticosteroid therapy from the physicians in charge and close follow-up of patients. Importantly, the synchronous administration of antifungal agents (e.g., fluconazole, posaconazole), which are frequently used in hematologic patients, may keep the axis suppressed for longer periods of time due to inhibition of the steroidogenesis pathway [102,103]. Nivolumab has been associated with adrenalitis and Addison’s disease [104], as well as hypothyroidism and secondary adrenal insufficiency due to selective pituitary dysfunction [105]. In addition, long-term survivors of childhood ALL, who were treated with moderate dose of cranial radiotherapy, exhibited central adrenal insufficiency 20 years after treatment [106].

### 3.6. Hyponatremia Related to Kidney Injury in Patients with Hematologic Diseases

Renal impairment affecting the urinary dilution capacity leads to hyponatremia due to decreased excretion of water. However, in less severe renal disease, water retention is limited and excessive water intake plays a major role in the development of hyponatremia [107]. Kidney disorders are often encountered in clinical practice. For example, acute kidney injury (AKI) due to prerenal azotemia may be induced by hemorrhage, gastrointestinal losses, renal losses (e.g., osmotic diuresis), low cardiac output, or decreased vascular resistance (e.g., due to infections). Of note, several pharmaceutical agents used in hematological disorders, such as platinum-containing drugs, alkylating agents, and methotrexate, are nephrotoxic [108,109]. Furthermore, contrast-induced kidney injury is common, especially in patients with cancer [110]. In a recent study, the incidence of AKI in hospitalized patients with hematological malignancies was 15.4% and serum sodium levels were directly related to AKI [111]. Hemoglobinopathies, complement disorders, and infections such as malaria may cause AKI via intravascular hemolysis [112]. Renal thrombotic microangiopathy leading to impaired renal function is a common complication associated with hematopoietic stem cell transplantation (HSCT) [113]. Kidney injury post-HSCT may also be the result of several other factors, including chemotherapy, radiation, sepsis, drugs (e.g., antibiotics, calcineurin inhibitors), bone marrow toxicity, hepatic veno-occlusive disease, and GvHD [114]. Renal impairment is very common in MM and other lymphoid malignancies and paraproteinemias [115,116,117]. Hypercalcemia, hyperuricemia, dehydration, renal parenchymal involvement, ureteral obstruction, glomerulonephropathy, renal vascular compromise, and tumor lysis syndrome may all be culprits of kidney injury in these patients [117]. In addition, patients suffering MM are prone to infections and often use non-steroid anti-inflammatory drugs (NSAIDs) as painkillers; these are also risk factors for AKI [118]. A case of cryoglobulinemic glomerulonephritis associated with nodal and renal infiltration by T-cell lymphoma of T-follicular helper phenotype has also been reported [119]. AKI is mainly observed in hemolytic uremic syndrome but may also occur in thrombotic thrombocytopenic purpura [120,121,122]. Paroxysmal nocturnal hemoglobinuria (PNH) may cause both acute and chronic renal impairment via several pathophysiological mechanisms, mainly including the release of free heme and iron due to intravascular hemolysis and subsequent hemoglobinuria, Fanconi syndrome, and possibly subclinical microvascular thrombosis [123].

Furthermore, chronic kidney disease is a common complication in sickle cell disease and sickle trait. This mainly occurs due to repetitive episodes of sickling, which induce ischemic damage and microinfarctions, leading to loss of the vascular architecture of the renal medulla. Other contributory factors are glomerular hyperfiltration and renal hyperperfusion, endothelial dysfunction, and the release of free heme due to hemolysis [124,125,126].

### 3.7. Hyponatremia Related to Cardiac Disorders in Hematologic Patients

Hyponatremia in heart failure (HF) is mainly attributed either to neurohormonal activation due to effective circulating volume depletion or to diuretic administration [127].

HF is a common complication of various hematologic diseases and drugs used in clinical hematology. Hemoglobinopathies, mainly beta (β)-thalassemia and sickle cell disease, are associated with HF in the context of chronic hemolysis and sickling. Specifically, chronic hemolysis leads to anemia and, accordingly, high-output HF, as well as vasculopathy, while sickling induces both vasculopathy and myocardial ischemia. Furthermore, repetitive blood transfusions and increased iron absorption from ineffective erythropoiesis in patients with hemoglobinopathies may induce iron overload cardiomyopathy [128]. Deposition of amyloid and iron in the heart in amyloid disease and hemochromatosis, respectively, have also been associated with cardiomyopathy [129].

Of note, the treatment of several hematological diseases may impair cardiac function. In particular, thoracic radiation may provoke cardiomyopathy 5 to 30 years after initial exposure [130], whereas anthracyclines have been shown to cause irreversible dose-dependent cardiac damage and late-onset HF [131]. Several studies have demonstrated the involvement of anthracyclines in the development of HF in survivors of both childhood- and adult-onset cancers, as well as in patients with HL or aggressive NHL [132,133,134,135]. Alkylating agents, such as cyclophosphamide, fluoropyrimidines, and tyrosine kinase inhibitors, have been associated with cardiovascular complications including HF [136,137,138]. Chimeric antigen receptor (CAR) T-cell therapy may have deleterious effects on the myocardium and even lead to HF via excessive release of cytokines [139]. Furthermore, immune checkpoint inhibitors have been associated with myocarditis [140].

### 3.8. Hyponatremia Related to Liver Diseases in Hematologic Patients

Hyponatremia is frequently encountered in hepatopathies, mainly cirrhosis, in part due to reduced effective arterial blood volume as well as other involved mechanisms. It should be noted that marked hyperlipidemia (hypertriglyceridemia and/or hypercholesterolemia) and hypergammaglobulinemia, which are also common features of liver disorders, are among the major culprits of pseudohyponatremia [37]. Liver involvement may be observed in the course of several hematologic diseases, potentially leading to or aggravating hyponatremia. Cirrhosis may be the outcome of hemosiderosis due to transfusional iron overload in beta (β)-thalassemias. In sickle cell disease, repeated episodes of vascular occlusion affecting the liver may impair its function. Autoimmune hemolytic anemia and autoimmune hepatitis may co-exist. Hepatic vein obstruction (Budd–Chiari syndrome) may occur in polycythemia vera or other myeloproliferative diseases, PNH, as well as in patients who have undergone bone marrow transplantation [141,142,143]. Liver infiltration (e.g., lymphoma, leukemia, multiple myeloma) may end up in a broad spectrum of disorders ranging from asymptomatic elevation of liver function tests to acute hepatic failure [144]. Importantly, certain diseases (e.g., hemochromatosis, Wilson disease) may have both hepatic and hematologic manifestations, whereas several comorbidities or sequelae of hematologic diseases (e.g., DM, HF, HBV, HCV) are implicated in the development of hepatic cirrhosis. Specifically, cirrhosis may be the result of DM-related non-alcoholic fatty liver disease, while HF may lead to hepatic fibrosis (“cardiac cirrhosis”) [37]. Furthermore, HBV and HCV infections after multiple blood transfusions in hematologic patients may also develop cirrhosis.

### 3.9. Hyponatremia Related to Pharmacological Agents Used in the Treatment of Blood Diseases

Many drugs commonly used for the treatment of certain hematological diseases have been associated with low serum sodium concentration (Table 1). The most common mechanism of drug-induced hyponatremia is SIADH. Nausea, a common side effect of chemotherapeutic agents, is a very potent trigger for ADH secretion. Vinca alkaloids, such as vincristine, induce hyponatremia through SIADH [145,146,147]. We should note that the simultaneous administration of vincristine with azoles is contraindicated, as the latter inhibit vincristine metabolism and, subsequently, may exacerbate the adverse effects of this drug, including neurotoxicity and hyponatremia [148,149]. CD19 + CAR T-cells used for the treatment of relapsed/refractory ALL may cause hyponatremia due to hypercytokinemia and elevated IL-6, which stimulates the hypothalamus for the inappropriate release of ADH [150]. Methotrexate in high doses may lead to hyponatremia, probably due to the toxic effects on the neurosecretory areas of the cerebrum, activation of natriuretic peptides, or changes in the distribution of body fluid volumes [109,151]. Cyclophosphamide causes hyponatremia, mainly when it is administered in high doses due to concurrent administration of large volume of hypotonic fluids in order to prevent hemorrhagic cystitis. However, hyponatremia may also be observed when cyclophosphamide is administered at lower doses [152]. It should be noted that several chemotherapy protocols mainly for patients with lymphoma, such as hyper-CVAD (cyclophosphamide, vincristine sulfate, doxorubicin hydrochloride, dexamethasone, methotrexate, cytarabine) and CODOX-M/IVAC (cyclophosphamide, vincristine, doxorubicin, high-dose methotrexate/ifosfamide, etoposide, high-dose cytarabine) include the co-administration of cyclophosphamide and vincristine. Both regimens carry a > 20% risk of febrile neutropenia, and antifungals such as azoles are routinely co-administered, increasing the risk of hyponatremia, as mentioned above. Selinexor, an oral selective inhibitor of nuclear export currently in clinical development for relapsed refractory multiple myeloma, has been shown to cause hyponatremia in almost 30% of patients [153]. Ibrutinib, which is widely used for the treatment of chronic lymphocytic leukemia, mantle cell lymphoma and WM, has been shown to cause hyponatremia in up to 6% of treated patients [154].

Platinum-based antineoplastic drugs may also lower sodium levels, mainly due to the concurrent administration of large volumes of hypotonic fluids in order to prevent nephrotoxicity [109]. Both SIADH and CSWS have been recognized as underlying mechanisms of hyponatremia in patients taking cisplatin [145,155,156]. In contrast, hyponatremia is less frequently encountered with oxaliplatin compared with cisplatin [157]. An increase in urinary *N*-acetyl β-glycosaminidase, a proximal tubule lysosomal enzyme, within 24–48 h after administration of cisplatin, is proposed as a predictor of developing hyponatremia associated with this drug [155,158].

Intravenous administration of sucrose-containing immunoglobulin may cause true hyponatremia attributed to the translocational effect of the osmotic load of sucrose [159]. Translocational (hyperosmolar) hyponatremia may also be observed with maltose-containing IVIG in the presence of renal impairment. In this setting, maltose, normally metabolized by maltase at the proximal renal tubules, is accumulated in the extracellular fluid, thus increasing serum osmolality and diminishing serum levels by means of dilution. Aseptic meningitis-associated SIADH is another potential underlying mechanism of hyponatremia following IVIG [39]. Most tyrosine kinase inhibitors (TKIs), specifically imatinib, nilotinib, dasatinib, and bosutinib have been dose-dependently associated with hyponatremia, mainly due to SIADH [160,161]. In contrast, ponatinib has not been associated with hyponatremia so far.

Drugs used in clinical hematology and their associated underlying mechanisms for hyponatremia are shown in Table 2.

Patients treated for hematological disorders are often administered several drugs for various other conditions, including tricyclic antidepressants, selective serotonin re-uptake inhibitors, proton pump inhibitors, antiepileptic drugs, trimethoprim-sulfamethoxazole, NSAIDs, tramadol, and other opioid analgesics, all of which have also been related to hyponatremia [39,162].

## 4. Evaluation of Hyponatremia

**Figure 1:**
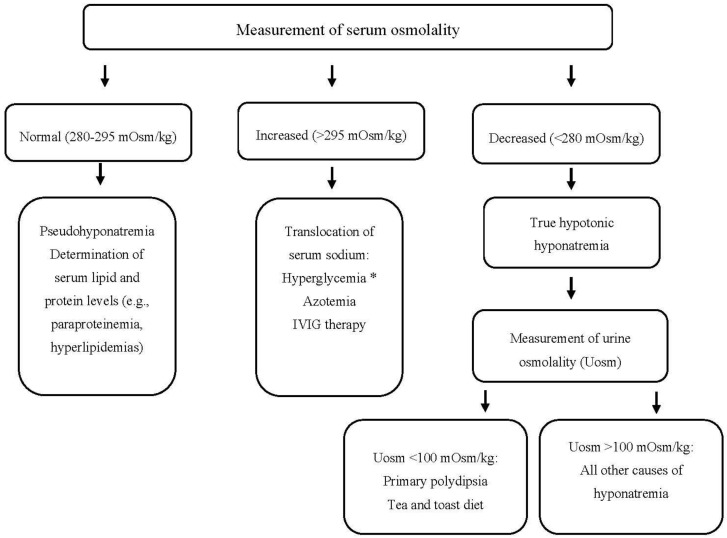
Approach to a patient with hyponatremia (and hematologic disease). * Serum sodium should be corrected for the degree of hyperglycemia. IVIG: Intravenous Immunoglobulin, mOsm: milliosmole, Uosm: urine osmolality.

Patients with hyponatremia frequently manifest severe neurologic complications due to brain edema. On the other hand, the rapid correction of hyponatremia may lead to the development of central demyelinating lesions, particularly in the pons (a disorder called central pontine myelinolysis or osmotic demyelination syndrome (ODS)) with major neurologic disabilities or even death [181]. Consequently, the timely recognition of the underlying cause(s) of hyponatremia is crucial for the appropriate management and avoidance of therapeutic pitfalls, which can lead to under- or over-treatment of hyponatremia. A step-by-step diagnostic evaluation of hyponatremia in hematologic patients is shown in Figure 1. Some points, however, deserve emphasis.

Urine sodium concentration (UNa) in a random urine specimen has a pivotal role in the diagnostic approach. Values less or more than 30 mEq/L are suggestive of low effective arterial blood volume or SIADH, respectively. However, UNa levels > 30 mEq/L are also found in case of diuretic administration, osmotic diuresis, salt losing nephropathy, primary adrenal insufficiency, and metabolic alkalosis. On the other hand, UNa < 30 mEq/L can be observed in patients with chronic SIADH on a low salt diet or anorexia. In the presence of metabolic alkalosis, a low urine chloride concentration (<25 mEq/L) is a useful index of extracellular volume depletion [37].

Hyponatremia due to SIADH and CSWS may be observed in hematologic diseases with intracranial involvement [182]. The latter has a similar laboratory picture with SIADH (i.e., hypouricemia along with a fractional excretion of uric acid (FEUA) > 11%, Una > 30 mmol/L and Uosm > 100 mOsmol/kg). Importantly, though, the treatment is not the same due to the different volume status in these disorders (i.e., normovolemia in the case of SIADH versus hypovolemia in the case of CSWS). The differentiation between CSWS and SIADH is often challenging in clinical practice, since the assessment of extracellular volume, not infrequently, is unreliable on clinical grounds. In fact, clear evidence of volume depletion (e.g., hypotension, decreased skin turgor, elevated hematocrit, increased blood urea nitrogen to creatinine ratio) is frequently absent in CSWS. Thus, in a seemingly normovolemic patient with hyponatremia associated with “intracranial disease”, fluid restriction, loop diuretics, and vaptans (ADH antagonists) used for the treatment of SIADH should be avoided, as they may deteriorate both hypovolemia and hyponatremia, leading to cerebral edema and even seizures in case of CSWS [183]. On the contrary, hypertonic saline should be administered in such cases. The estimation of FEUA after correcting hyponatremia is considered a useful tool in establishing the correct diagnosis given that FEUA normalizes in SIADH but remains > 11% in CSWS. Noteworthy, isotonic saline may aggravate hyponatremia due to SIADH if the Uosm is higher than serum osmolarity (especially in cases of Uosm > 530 mOsm/kg), thus, it should be avoided [182,184].

Hypopituitarism with secondary adrenal insufficiency is another overlooked cause of hyponatremia often presenting with a SIADH-like picture (euvolemic hyponatremia, low serum uric acid, and urea levels, UNa > 30 mmol/L, Uosm > 100 mOsmol/kg). Moreover, the differentiation between primary and secondary adrenal insufficiency are not infrequently difficult given that typical characteristics ascribed to mineralocorticoid deficiency, may not be present in the latter. Indeed, hyperkalemia may be absent in approximately 30–50% of patients with Addison’s disease, while hypovolemia as clinically assessed may not be evident. In such cases, certain diagnostic tests (cortisol determination and adrenocorticotropic hormone (ACTH) stimulation test) may be required for the diagnosis of hypothalamic–pituitary–adrenal axis disorders [16].

**Figure 2:**
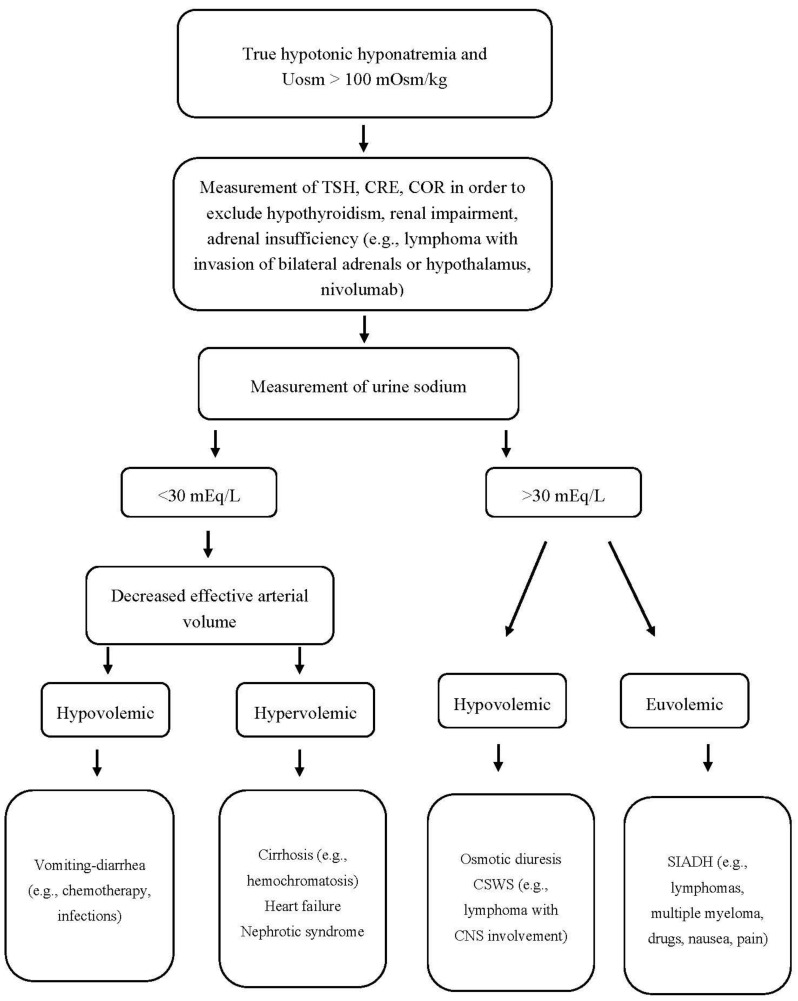
Approach to a patient with hypotonic hyponatremia, Uosm > 100 mOsm/kg, and hematologic disease. Uosm: urine osmolality, TSH: thyroid-stimulating hormone, CRE: creatinine, COR: cortisol, CSWS: cerebral salt wasting syndrome, SIADH: syndrome of inappropriate antidiuretic hormone secretion, CNS: central nervous system.

Finally, it should be pointed out that hypothyroidism-induced hyponatremia is rather rare and probably occurs only in severe hypothyroidism (TSH > 50 mIU/L). Even in myxedema coma, however, other possible causes and superimposed factors of hyponatremia (e.g., drugs, infections, adrenal insufficiency) should be considered before attributing the low serum sodium levels to hypothyroidism per se [185]. An algorithm for the proper approach to a patient with hypotonic hyponatremia, Uosm > 100 mOsm/kg, and hematologic disease is shown in Figure 2.

## 5. Treatment of Hyponatremia

Treatment of hyponatremia is mainly focused on how to avoid the devastating neurologic complications, which may occur either during the course of or after overcorrection of this electrolyte disorder. The appropriate rate of correction of hyponatremia should be <8–10 mEq/L/24 h [181,186]. However, in the presence of conditions that predispose patients to develop ODS (i.e., hypokalemia, malnutrition, liver disease, alcoholism, and serum sodium levels ≤ 105 mEq/L), the appropriate rate of correction should be limited to 4–6 mEq/L/24 h [181,186,187]. As a general rule, the therapeutic interventions for hyponatremia are based on its duration, as well as on patient’s symptoms and extracellular volume status [18,188].

In cases of severe neurological symptoms due to hyponatremia a bolus infusion of 100–150 mL of hypertonic saline (3% NaCl) over 20 min, up to three times is recommended [186,187]. The goal is an increase in serum sodium concentration by 4–6 mEq/L within the first 4–6 h in order to reverse the symptoms of hyponatremic encephalopathy, without, however, exceeding the aforementioned targets. Continuous infusion of 3% NaCl (0.5–2 mL/kg/hour) can be used for moderate symptoms [186,187,189,190].

Treatment of the underlying conditions related to hyponatremia (e.g., infections, hyperglycemia, primary adrenal insufficiency) and discontinuation of diuretics are crucial in the management of hypovolemic hyponatremia. Normal saline or lactated Ringer’s solution should be used to restore the intravascular volume [187,191]. Close monitoring of serum sodium levels (every few hours) and urine output is strongly recommended in order to avoid an overly rapid increase in sodium concentration when volume status is restored. In this setting, an abrupt decrease in ADH secretion and, subsequently, a rapid increase in diuresis is observed. Noteworthy, in hypovolemic states, any potassium deficit should also be corrected. In such cases, potassium chloride should be added in hypotonic fluids. The administration of normal saline plus potassium chloride (i.e., a hypertonic solution) should be avoided, as it increases the risk of overcorrection of hyponatremia as well as that of volume overload and pulmonary edema, especially in the elderly or in patients with HF [191].

Fluid restriction is the first line therapy in hyponatremia due to SIADH. Discontinuation of the offending drugs and treatment of other superimposed factors (e.g., infection, pain, nausea) are also essential. Increased solute intake (such as salt tablets or urea) and loop diuretics can also be used to increase water clearance.

In contrast, restriction of fluid intake and administration of loop diuretics to correct increased total body water and sodium are required for the treatment of hypervolemic hyponatremia. Vaptans—drugs which promote water diuresis—are indicated as second-line therapy for hyponatremia related to hypervolemic or euvolemic hyponatremia due to SIADH [188]. Importantly, vaptans should not be used in hypovolemic hyponatremia or together with hypertonic saline solution owing to case reports of associated ODS [186,187,192]. Edematous hyponatremia, in the context of severe acute or chronic kidney injury generally, requires dialysis. Basic principles of hyponatremia management are shown in Table 3.

## 6. Conclusions

Hyponatremia, often multifactorial, is frequently observed in patients with hematologic disorders and may aggravate their already fragile clinical condition. The severity of this disorder is often underestimated because of its non-specific clinical features that may be mistakenly attributed to other clinical entities, such as neutropenic sepsis, chemotherapy side effects, CNS involvement, or disease-related fatigue. Special efforts should be made to define the underlying causal mechanisms and appropriately individualize therapy. Specific attention should be given to the co-administration of drugs that alter each other’s metabolism, as this may contribute to or aggravate an already existing hyponatremia. Beyond the specific treatment of hyponatremia based on its duration, symptoms, and extracellular volume status, the therapeutic approach should involve discontinuation of any offending medications and management of other conditions (e.g., infectious, cardiac, renal, or endocrine) that may also contribute to this disorder. Overall, it is important for hematologists to become familiar with the timely recognition and appropriate therapeutic approach of hyponatremia.

## Figures and Tables

**Table 1:** Causes and/or superimposed factors of hyponatremia in hematological patients.

Type of Hyponatremia	Causes	Associated Conditions
Pseudohyponatremia (isotonic hyponatremia: serum osmolality 280–295 mOsm/kg)	Hyperglobulinemia	MM, other paraproteinemias, POEMS syndrome, Castleman’s disease, post-transplant monoclonal gammopathies, NHL, CLL, cryoglobulinemia, cold agglutinin disease, Gaucher disease, HCV or HIV infection, cirrhosis drugs: IVIG, interferon.
	Hypertriglyceridemia	HLH, uncontrolled diabetes mellitus drugs: L-asparaginase, ATRA. Interferon.
	Hypercholesterolemia	Allogeneic stem cell transplantation, MM, NHL.
	Mixed dyslipidemia	Nephrotic syndrome (secondary causes include: HL, monoclonal gammopathy, cryoglobulinemia, POEMS syndrome, leukemia, glycogen storage diseases, sickle cell disease, MDS, GVHD, infections).
Hypertonic hyponatremia: (serum osmolality >295 mOsm/kg)	Hyperglycemia	Diabetes mellitus: preexisting or related to hemochromatosis, thalassemia, and HSCT. Infections. Drugs: glucocorticoids, interferon, tacrolimus, immune checkpoint inhibitors.
Hypotonic hyponatremia (serum osmolality <280 mOsm/kg)	Hypervolemic (edematous hyponatremia)	Cirrhosis. Nephrotic syndrome. Renal insufficiency: chemotherapy, contrast media, NSAIDs, infections, post-HSCT, PNH, sickle cell disease and other hemoglobinopathies, MM, lymphomas, paraproteinemia, TTP, HUS, TLS Heart failure: cardiomyopathy due to hemochromatosis, hemoglobinopathies, amyloidosis, or paraproteinemias. Drug-induced heart failure (e.g., anthracyclines, alkylating agents, fluopyrimidines, TKIs), CAR T- cell therapy, thoracic radiation therapy, immune checkpoint inhibitors-induced myocarditis. POEMS syndrome. (Extravascular volume overload is among the minor criteria of the syndrome).
	Euvolemic hyponatremia	SIADH: lymphomas, paraproteinemias, HLH, post-HSCT, sickle cell disease, porphyria. Pain and nausea. Pulmonary and CNS infections. Drugs: vinca alkaloids, cyclophosphamide, platinum compound drugs, melphalan, busulfan, interferon, bortezomib, TKIs, methotrexate, tacrolimus, cyclosporine A, CAR T-cells therapy, carbamazepine, SSRIs.
	Hypovolemic hyponatremia	Adrenal insufficiency: adrenal metastases or CNS invasion by lymphomas, AML, ALL, hemoglobinopathies. Infections: CNS infections (e.g., tuberculosis), systemic fungal infections. Bilateral cranial radiation therapy. Drugs: steroids, immune checkpoint inhibitors. Extra-renal salt loss: infection mediated. Renal salt loss: myeloproliferative diseases, LAHS, sickle cell disease. Post-HSCT with CNS complications. Drugs: methotrexate, hydroxyurea, platinum compounds, etc. Adrenal hemorrhage: e.g., coagulopathy.

ALL: acute lymphoblastic leukemia, ATRA: all trans retinoic acid, AML: acute myeloid leukemia, CAR: chimeric antigen receptor, CML: chronic myeloid leukemia, CLL: chronic lymphocytic leukemia, CNS: central nervous system, GVHD: graft versus host disease, HCV: hepatitis C virus, HIV: human immunodeficiency virus, HL: Hodgkin’s lymphoma, HLH: hemophagocytic lymphohistiocytosis, HSCT: hematopoietic stem cell transplantation, HUS: hemolytic uremic syndrome, IVIG: intravenous immunoglobulin, LAHS: lymphoma-associated hemophagocytic syndrome, MDS: myelodysplastic syndrome, MM: multiple myeloma, NHL: non Hodgkin, POEMS: polyneuropathy, organomegaly, endocrinopathy, monoclonal gammopathy, and skin changes, SSRIs: selective serotonin reuptake inhibitors.

**Table 2:** Drugs used in clinical hematology and associated underlying mechanisms for hyponatremia.

Drug	Disease	Mechanism
Cyclophosphamide, ifosfamide [152,163,164,165]	Multiple myeloma, lymphomas	SIADH, hypotonic fluids to prevent hemorrhagic cystitis
Imatinib and other TKIs [160,161]	CML, ALL	SIADH
Entospletinib [166]	In development	SIADH
Vincristine [145,146,147]	Lymphomas, ALL	SIADH
Methotrexate [167]	Lymphomas, ALL	SIADH and CSWS
mTOR inhibitors (everolimus) [168]	In development	Aldosterone resistance
Tacrolimus [56]	Post AlloSCT	SIADH
Selinexor [153]	In development	Unknown
Cytarabine, Elacytarabine [169]	Lymphomatous meningitis, AML	Unknown
Hydroxyurea [170]	Myeloproliferative neoplasms, sickle cell disease	CSWS
Ibrutinib [154]	CLL, mantle cell lymphoma, Waldenstöm’s macroglobulinemia	Unknown
Nivolumab, Pembrolizumab [104,171,172]	Hodgkin lymphoma	Primary adrenal insufficiency, Diabetes mellitus
Rituximab plus lenalidomide [173]	Follicular lymphoma	Unknown
Pentostatin [174]	HCL	Unknown
Cyclosporine A [175]	Immunosuppression post AlloSCT	SIADH
Desmopressin [176,177]	Bleeding disorders	Unknown
Intravenous immunoglobulin (IVIG) [159]	ITP	Translocational effect of the osmotic load of sucrose and IVIG nephropathy
Bortezomib [178]	Multiple myeloma	SIADH
CD19+ chimeric antigen receptor (CAR) T-cells [150]	ALL, DLBCL, PMBCL	Elevated IL-6→ SIADH
Platinum compounds (cisplatin, carboplatin, oxaliplatin) [145,155,156]	Relapsed or refractory lymphomas	SIADH, CSWS, hypotonic fluids
Interferon [179]	HCL, CML, multiple myeloma, follicular lymphoma	SIADH
Melphalan [180]	Multiple myeloma	SIADH
Busulfan [109]	Prior to SCT	SIADH

ALL: acute lymphocytic leukemia, AlloSCT: allogeneic stem cell transplantation, AML: acute myeloid leukemia, CML: chronic myeloid leukemia, CLL: chronic lymphocytic leukemia, CSWS: cerebral salt wasting syndrome, DLBCL: diffuse large B-cell lymphoma, HCL: hairy cell leukemia, ITP: immune thrombocytopenia mTOR: mammalian target of rapamycin, PMBCL: primary mediastinal B-cell lymphoma SIADH: syndrome of inappropriate secretion of antidiuretic hormone, TKIs: tyrosine kinase inhibitors.

**Table 3:** Basic principles of hyponatremia management.

Determination of time onset of hyponatremia (acute < 48 h and chronic > 48 h).
Proper correction rate < 8–10 mEq/L/24 h.
Proper correction rate of 4–6 mEq/L/24 h in high risk conditions for ODS. Hypokalemia, malnutrition, advanced liver disease, alcoholism, serum sodium ≤ 105 mEq/L.
In acute symptomatic hyponatremia, administration of hypertonic saline solution (3% sodium chloride) is prudent.
In patients with SIADH, fluid restriction, furosemide, or vaptans are the major treatment options.
In hypovolemic patients and CSWS, fluid restriction, furosemide, and vaptans are contraindicated. Instead, isotonic saline solution may be administered.

CSWS: cerebral salt wasting syndrome, h: hours, ODS: osmotic demyelination syndrome, SIADH: syndrome of inappropriate secretion of antidiuretic hormone.
